# Enthalpy *vs.* friction: heat flow modelling of unexpected temperature profiles in mechanochemistry of metal–organic frameworks†
†Electronic supplementary information (ESI) available. See DOI: 10.1039/c7sc05312f


**DOI:** 10.1039/c7sc05312f

**Published:** 2018-01-23

**Authors:** Krunoslav Užarević, Nenad Ferdelji, Tomislav Mrla, Patrick A. Julien, Boris Halasz, Tomislav Friščić, Ivan Halasz

**Affiliations:** a Division of Physical Chemistry , Ruđer Bošković Institute , Bijenička c. 54 , 10000 Zagreb , Croatia . Email: ivan.halasz@irb.hr ; Email: krunoslav.uzarevic@irb.hr; b Faculty of Mechanical Engineering and Naval Architecture , University of Zagreb , Ul. Ivana Lučića 5 , 10000 Zagreb , Croatia; c Department of Chemistry , McGill University , Montreal , H3A 0B8 Canada

## Abstract

Mechanochemical reactions by ball milling are becoming increasingly popular across a wide range of chemical sciences, but understanding and evaluation of temperature during such processes remains a persistent challenge, especially for organic and metal–organic materials. Here, we describe the first methodology for precise real-time measurement of sample temperature during mechanochemical transformations. Using this technique coupled with real-time *in situ* reaction monitoring by synchrotron X-ray diffraction and numerical simulations of heat flow, we have shown that the temperature profiles of mechanochemical reactions are dominantly determined by the energy dissipated through friction between the sample and the moving milling assembly, while the reaction enthalpy will usually be comparatively insignificant. With the changes in composition during mechanochemical reactions, frictional properties of the milled material change, leading to either better or worse energy absorption upon collisions in the process of milling. This approach explains unexpected and rapid temperature drops during exothermic transformations of ZIF-8 polymorphs. Since reaction kinetics are highly sensitive to changes in temperature, precise temperature profiles provided here will be mandatory to understand kinetics and its changes during milling, and will aid in developing the comprehensive model of mechanochemical reactivity.

## Abstract 2

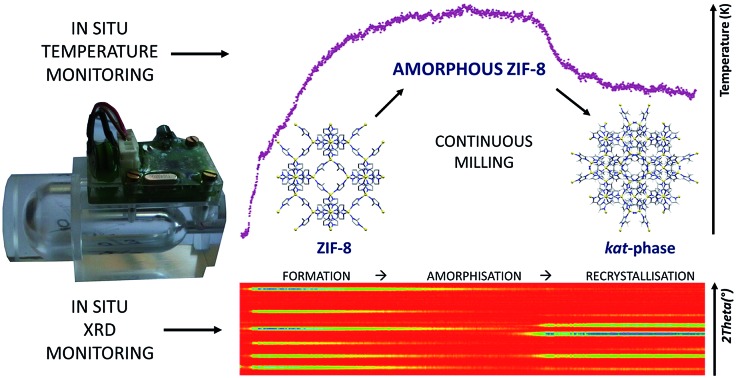
Numerical simulations for precise temperature profiles of milling reactions revealed dominant contribution of frictional heating, while reaction enthalpy remained negligible.

## Introduction

Mechanochemical solid-state reactivity^1^–^3^ offers advantages over traditional solvent-based chemistry reflected in better yields and selectivity,^4^,^5^ better energy- and atom-economy and reduced waste generation.^1^ Despite the longstanding use,^6^,^7^ range of applications, and industrial importance of mechanochemical reactions, an overarching mechanistic framework, which is a prerequisite for predictable and controllable mechanochemistry, has yet to be formulated.^8^–^10^ Recent reports on the sensitivity of mechanochemical reactions to heating^11^–^13^ as well as the observation of base-catalysis^14^ suggest that it should be possible to formulate a mechanistic framework for mechanochemical milling reactions, similar to the one existing for solution reactions developed by methods of physical chemistry in the better part of the 20^th^ century.^15^

Thus far, major theories of mechanochemical reactivity have focused on inorganic and metal systems and have ascribed mechanochemical reactivity to localised and sudden increases in temperature of the order of 10^3^ K at the points of ball impacts.^16^,^17^ However, there is growing evidence that these theories, which do not consider the temperature of the reaction mixture and the milling assembly, are not adequate^18^ for describing milling reactions of softer organic or metal–organic materials, for which recent evidence shows that even a modest increase in bulk temperature (by *ca.* 25–50 K) may lead to significantly faster reactions and changes to reaction mechanism.^18^ Despite recent significant advances in mechanistic understanding of mechanochemistry, the effect of temperature (one of the most basic factors for understanding kinetics and mechanisms in solution and gas chemistry) on mechanochemical milling reactions remains a challenge.

The study of kinetics and mechanochemical milling reaction mechanisms was virtually impossible until the recent development of techniques for *in situ* and direct monitoring of mechanochemical milling reactions through synchrotron powder X-ray diffraction (PXRD)^19^–^22^ or Raman spectroscopy.^23^,^24^ It was revealed that mechanochemical reactions that can proceed through crystalline or amorphous intermediate phases are strongly dependent on different types of additives, and can provide access to short-lived metastable intermediate phases that are inaccessible by other synthetic methods.^25^ Nevertheless, apart from temperature monitoring of highly-exothermic mechanochemical self-sustained reactions (MSR, exemplified by the “thermite” reaction),^26^–^29^ the effect of temperature on the reactivity of softer materials (*e.g.* cocrystals, metal–organic frameworks, coordination polymers) is far less understood.

Measuring and monitoring the temperature of a reaction mixture within the milling vessel is challenging, as a temperature sensor is likely to be broken under ball impacts and mill vibrations. Thus far, it has mostly been attempted for milling in steel milling vessel planetary ball mills.^31^,^32^ In MSR, the reaction-energy release is immense and sudden; consequently, temperature changes can be observed by attaching a sensor on the outside wall of the steel reaction vessel^27^,^33^ or by employing an infrared thermometer along with a quartz reaction vessel.^34^ More recently, an attempt has been made at monitoring the temperature by using infrared imaging.^35^ This method is limited to the outside surface of the reaction vessel and consequently, does not directly convey the temperature of the milled sample.

**Fig. 1:**
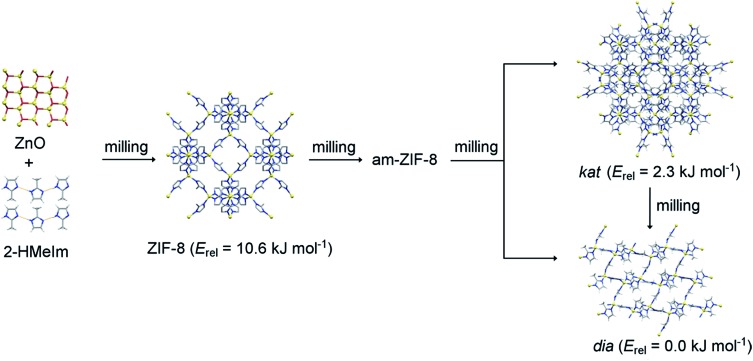
Reaction sequence of mechanochemical crystallisation–amorphisation–recrystallisation of ZIF-8. Relative energies (*E*_rel_) of ZIF-8 and its polymorphs are taken from ref. 30.

Here, we demonstrate the first methodology for the evaluation of temperature changes within an operating milling vessel. Using mechanochemical transformations of polymorphs of archetypal metal–organic frameworks (MOFs) such as the formation and mechanochemical transformations of ZIF-8 (Fig. 1) or pillared MOFs as model systems, and by conducting thermal measurements simultaneously with *in situ* synchrotron X-ray powder diffraction, here we have reported the first real-time temperature profiles for a mechanochemical reaction and have shown how they can be correlated to structural transformations of milled materials. Numerical simulations revealed that the measured temperature profiles are not significantly influenced by enthalpic changes of mechanochemical reactions, but are determined by changes in the frictional properties of materials in the reaction mixture. This provides a conceptually different way for thermal monitoring during the course of a mechanochemical reaction, which enables the detection of transformations even if the associated enthalpic change is small.

## Results and discussion

To enable reaction mixture temperature measurements, we have developed a reaction vessel using thermally insulating polymethylmethacrylate (PMMA, thermal conductivity ≈ 0.2 W m^–1^ K^–1^) (see ESI†) which contained a small aluminium plug (thermal conductivity ≈ 200 W m^–1^ K^–1^) embedded in the vessel wall and was in direct contact with the inside of the vessel. A temperature sensor (Pt100 sensor) was in contact with the aluminium plug ensuring a good thermal contact between the temperature sensor and the reaction mixture (ESI Fig. 1 and 2†). This setup avoided the low thermal conductivity of PMMA and achieved fast temperature readings with a precision within ±0.03 °C (ESI Fig. 3†).

Small temperature changes of the reaction mixture could be detected with a minimal time delay. However, we noted that due to localised ball impacts and having the sample in the form of a loose powder, the temperature of the sample might possibly be not uniform and variations in the sample temperature at different parts of the reaction vessel would not be revealed from this setup. Nevertheless, as far as we are aware, this represents the most precise and immediate temperature monitoring of mechanochemical reactions performed thus far.

As milling is initiated, temperature of the reaction mixture rises due to dissipation of the kinetic energy of the milling assembly into its internal energy. This process includes inelastic collisions between the milling media and the vessel walls, as well as the friction between the moving balls, the vessel, and the milled material. From now on, we shall refer to this overall process as friction.^36^ In parallel with energy input *via* friction, the reaction vessel constantly transfers energy to the cooler surroundings, which was air-conditioned at 20.5 °C. As the temperature of the milling assembly rises after initiation of milling, so does the rate of heat transfer to the surroundings. Eventually, when the rate of such heat transfer to the surroundings becomes equal to the energy dissipated by friction during milling, the system reaches a steady state in which the temperature does not change in time.

**Fig. 2:**
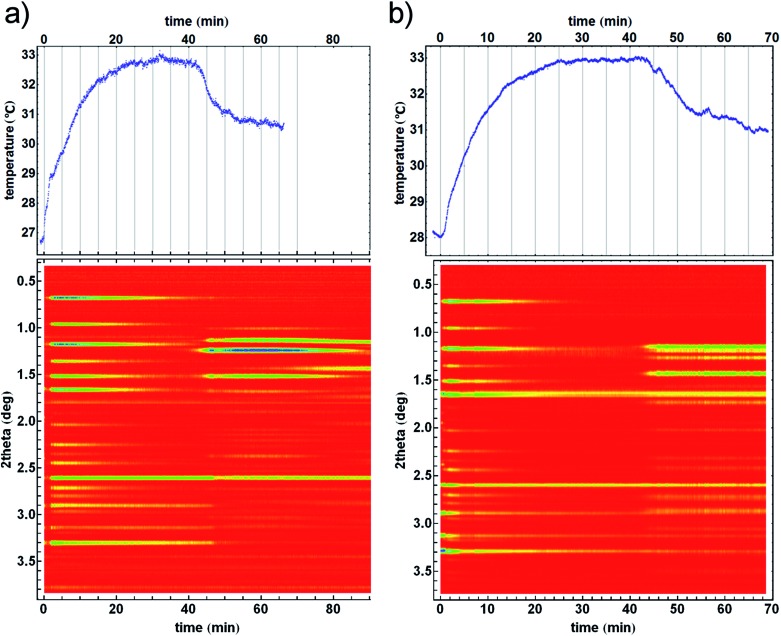
Time resolved diffractograms with their temperature profiles for crystallisation of the (a) *kat* polymorph of ZIF-8 from the amorphous matrix and (b) crystallisation directly into the *dia* polymorph. Homogeneity and uniformity of the reaction mixture is evident from the uniformity of the diffraction signal from crystalline silicon (at 2.6° in 2theta) which was used as an internal scattering standard.^22^

The first target in our study was the recently reported mechanochemical crystallization–amorphization–recrystallization of the zeolitic imidazolate framework zinc 2-methylimidazolate (Zn(**MeIm**)_2_), also known as ZIF-8, by liquid-assisted grinding (LAG) with dilute aqueous acetic acid as the liquid additive (Fig. 1).^25^ The ZIF-8 framework is one of the few commercially relevant MOFs, and has received attention for its chemical and thermal stability, as well as for its applications in gas storage and catalysis.^37^–^44^ Also, it was often used as a model system in mechanochemical MOF amorphisation,^45^ MOF alloying,^46^ and shock dissipation.^47^ As previously reported,^25^ milling of ZnO with 2-methylimidazole (**HMeIm**) leads to rapid formation of ZIF-8, which gradually becomes amorphous upon milling. Herein measured *in situ* X-ray diffraction data show that upon extended milling, the amorphous matrix (am-ZIF-8) recrystallizes into one of two Zn(**MeIm**)_2_ polymorphs. That is, recrystallization can yield either a metastable phase with katsenite (*kat*) topology,^25^ which upon further milling transforms into the thermodynamically stable, non-porous diamondoid (*dia*) topology polymorph^48^ (Fig. 2a, bottom). Alternatively, the *dia*-framework can also be formed directly^25^ from the amorphous matrix, circumventing the intermediate formation of the *kat*-phase (Fig. 2b, bottom).

The observed structural transformations of Zn(**MeIm**)_2_ frameworks could also be detected through temperature measurements. Formation of ZIF-8 from ZnO and 2-methylimidazole was very fast, and was accompanied by a steep increase in temperature during the first two minutes of milling. The formation of ZIF-8 was rapidly followed by its amorphisation, and at the same time the heating curve reached a steady-state temperature just below 33 °C, as the amorphisation process was complete. Recrystallisation of amorphous ZIF-8 manifested itself with the emergence of new Bragg reflections, and the temperature of the reaction mixture simultaneously dropped by *ca.* 2.5 °C over a period of *ca.* 15 minutes, after which it reached a new steady-state temperature just below 31 °C (Fig. 2a, top). In another experiment, the amorphous phase recrystallised directly to the *dia* ZIF phase, resulting in a *ca.* 2 °C temperature drop of the reaction mixture (Fig. 2b, top). Importantly, in both experiments, the temperature drop was directly correlated with the recrystallization of the amorphous Zn(**MeIm**)_2_.

The observed drops in temperature are surprising, since the observed polymorphic transformations should be exothermic. According to the recent calorimetric and theoretical studies, the close-packed *dia* polymorph is the most stable among the three polymorphs.^30^ The enthalpies of formation of the ZIF-8 and *kat* polymorph are 10.6 kJ mol^–1^ and 2.3 kJ mol^–1^, respectively, which are smaller than that of the *dia* polymorph. In accordance with the Ostwald's rule of stages, am-ZIF-8 has the energy of formation between that of ZIF-8 and the *kat* polymorph. Also, measured enthalpy of formation of ZIF-8 (ref. 30) corresponded to the empty-pore ZIF-8 while here, its pores were filled by the liquid additive leading to stabilisation of ZIF-8.

For the employed scale of the reaction (196 mg of the reaction mixture or 0.80 mmol of ZnO and 1.6 mmol of **HMeIm**), these energy differences should produce a net energy release on the order of 10 J. Over a period of *ca.* 30 minutes, which is the duration of amorphization and recrystallization to the *kat* polymorph and the final *dia*-phase formation, this amount of energy would generate a heat flow of around 3–6 mW. Polymorphic transformations in this system were expected to lead to a temperature increase, rather than the experimentally observed temperature drop. Moreover, if the temperature drop was related to some endothermic process, the constant input of energy by milling should have resulted in the return of the temperature to the previous steady-state after the endothermic process has ended, as was generally observed for MSR reactions.^27^

Clearly, both the conversion of mechanical energy of the vibrating milling assembly into its internal energy, and the reaction enthalpy can cause a temperature change in the vessel. The main difference is that milling produces a continuous heat flow *via* friction during the entire milling process, while the reaction energy release/absorption is limited and occurs only over a relatively short period of time. To understand the observed temperature profiles and heat flow during mechanochemical processing, we applied the First law of thermodynamics:

1 *Q*_friction_ + *Q*_reaction_ = *U*_2_ – *U*_1_ + *Q*_out_

where *Q*_friction_ corresponds to heat input *via* conversion of mechanical energy, *Q*_reaction_ is heat input from the reaction enthalpy, and *Q*_out_ is heat output to the environment. The difference between heat input and output results in the change of the internal energy of the milling assembly in time: *U*_2_ – *U*_1_.

The above equation is valid for any period of time. Since the friction heat is of rapid stochastic nature (a large number of isolated impacts in time), if we use its mean value over a reasonably short time interval and consider it as a continuous variable, we can write the equation for the infinitesimally short period of time:2
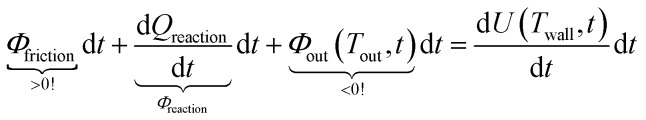



Integration over the entire duration of the reaction reveals that the second term on the left is constant, as it occurs over a limited period of time and therefore becomes increasingly negligible compared to the other two constantly increasing terms as the integration time becomes longer and the heat exchange approaches its steady state:3
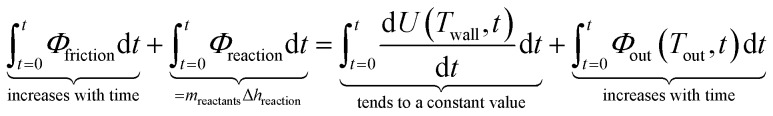



Heat flow generated by friction, *Φ*_friction_, is temperature- and time-independent if the milling parameters including the milled material remain unchanged, while heat transfer to the environment, *Φ*_out_ depends on the temperature difference between the outer surface and the ambient environment, and increases as the whole milling assembly warms up. A more elaborate analysis of energy conversion and heat transfer during milling is given in the Experimental section.

If milling proceeds with no chemical reaction or polymorphic transformation, temperature profile should exhibit monotonous heating until a steady state is reached. Indeed, this is observed in the temperature profile of an empty vessel (containing milling balls but without any material) and in the temperature profile of milling of pure ZnO, which was one of the reactants in the studied ZIF transformations (ESI Fig. 4 and 5†). An empty vessel heats up slower than a vessel containing ZnO, which is explained by the better absorption of kinetic energy of the milling balls upon collisions when some material is present in the reaction vessel. Difference in energy absorption was verified by a bouncing test where a steel ball was dropped onto a PMMA plate and the height of its bounce was measured. It was revealed that balls bounce from the PMMA plate with 0.86 coefficient of restitution (the ratio of the bounced height and the initial height from which the ball was dropped). On the other hand, a ball bounced on the same PMMA surface but now lined with a 1 mm thick layer of ZnO powder resulted in a less elastic collision (0.3 coefficient of restitution), indicating a more efficient conversion of the kinetic energy of the ball into the internal energy of the system. In support of such interpretation, significant heating of the mixture was observed when soft milling balls were used instead of hard ones. Soft milling balls were able to deform and thus efficiently dissipated their kinetic energy resulting in a temperature rise of over 100 °C.^49^

Characteristics of collisions between milling balls and the vessel walls thus determine the amount of the dissipated energy by friction during ball milling, which causes an increase in temperature of the milling assembly. To gain a quantitative insight into temperature profiles and heat exchange, we have performed numerical simulations of heat transfer between the milling assembly and the environment during milling. Energy dissipation from milling ball impacts was described as a heat source on the whole inside surface of the milling vessel, in accordance with the assumed random motion of milling balls.

At the start of the simulation, the heat flow immediately increased the temperature of the inside vessel surface. The heat was then conducted through vessel walls towards the environment. While energy dissipation remained constant throughout milling, heat flow to the environment increased with the increase in temperature of the whole milling assembly, finally reaching a steady state when the heat loss equalled the input energy dissipation. In our simulations, we have modified energy dissipation for each experiment in order to reproduce the observed temperature profiles. This has enabled us to estimate the energy dissipation for an empty vessel to be 272 mW while it was estimated to be 496 mW for the vessel containing ZnO.

**Fig. 3:**
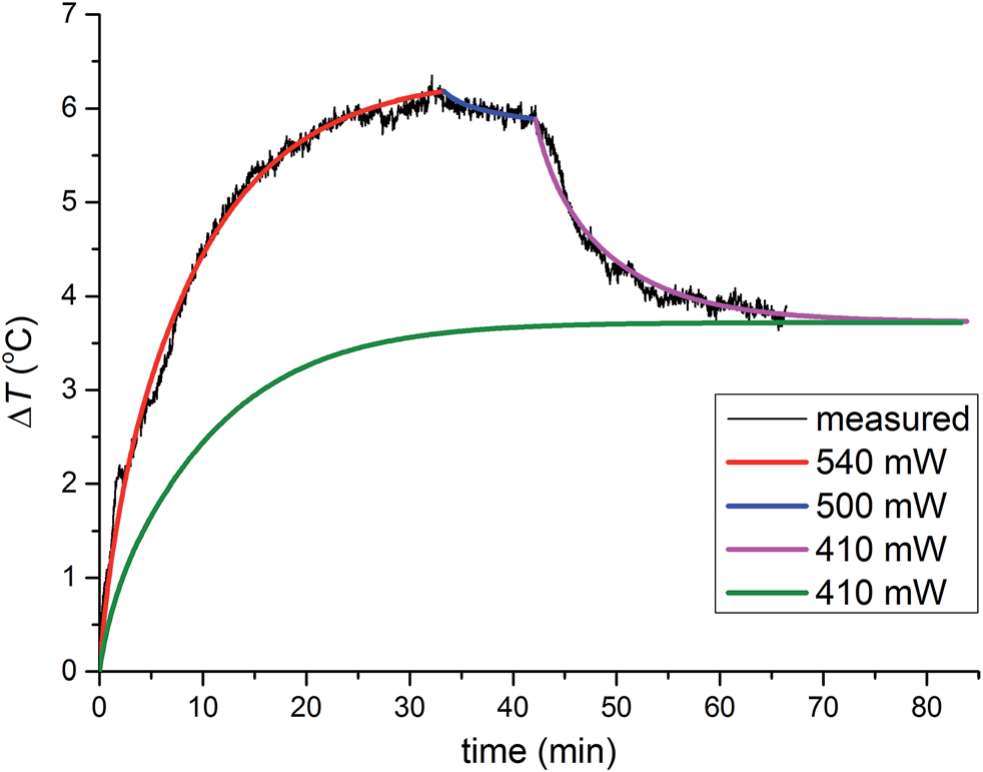
Numerical modelling of temperature profiles. Three-stages of the reaction: formation of ZIF-8, its amorphisation and finally, recrystallisation of the amorphous matrix into the *kat* phase are depicted with red, blue and purple curves, respectively (from Fig. 2a). The heat flow rate (in milliwatts) is divided into sections where each section corresponds to a specific reaction mixture composition. After recrystallisation to the *kat* phase, the heat flow rate dropped from 500 mW to 410 mW. In a hypothetical milling experiment using identical ambient conditions, the same steady-state temperature was reached by using heat flow rate of 410 mW from the beginning (green curve).

Constant energy dissipation leads to a monotonous temperature increase and a steady state temperature. However, more complicated temperature profiles which involve chemical changes, required modelling of heat flow in each stage of the reaction. During ZIF-8 formation and amorphization (Fig. 3), the heat flow upon 33 minutes of milling amounted to *ca.* 540 mW. A short period followed when the heat flow was reduced to *ca.* 500 mW and the final period after recrystallisation to the *kat*-phase, when the heat-flow rate was further reduced to *ca.* 410 mW.

The milling reaction was thus separated into three periods that have different friction properties generating different heat flows and consequently, leading to different steady-state temperatures. To illustrate that the steady-state temperature under the same ambient conditions is determined only by frictional properties of the material in the vessel and the associated rate of heat flow, this three-stage simulation was compared to a simulation of a putative milling process in which the frictional properties were immediately set to be identical to those of the third stage of the reaction, with the corresponding heat flow rate of 410 mW. This simulation resulted in a significantly lower temperature profile but reached the same steady-state temperature, confirming that the steady-state temperature under unchanged ambient conditions is determined by a permanent heat source inside the vessel.

The steady-state temperature depends on the rate of heat flow generated by the milling process and according to the Newton's law of cooling on ambient conditions. At the same time, the effect of any short-term heating events *e.g.* reaction-related release or absorption of energy, is expected to diminish over time. Thus, contrary to recent suggestions^35^,^52^ it is not possible to directly compare the temperature profile for milling of one inert material, to the profile of milling another reactive material, as these are characterized by very different frictional properties. Consequently, it is also not possible to interpret any differences between such temperature profiles in terms of reaction enthalpies. A more detailed account of heat exchange of the milling process is given in the ESI.†

**Fig. 4:**
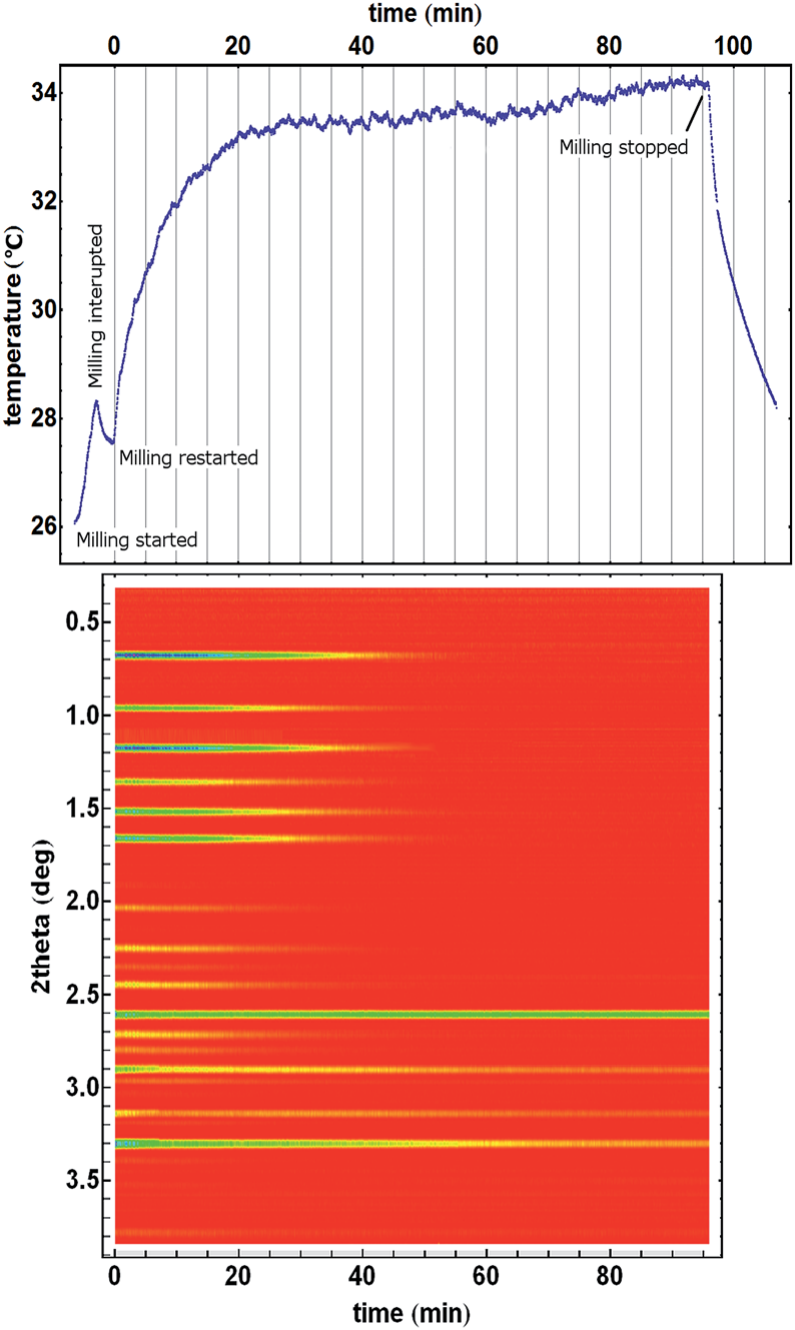
Amorphisation of ZIF-8 without recrystallization. Milling was started but had to be interrupted to restart diffraction data collection. The temperature profile first shows heating, followed by a cooling step before another onset of heating after milling was restarted. ZIF-8 is already present and the mixture seems homogenous at the beginning of PXRD monitoring because of the first interrupted milling period. After milling was stopped, the reaction vessel spontaneously cooled as per Newton's law of cooling. Homogeneity and uniformity of the reaction mixture is evident from the uniformity of the diffraction signal from crystalline silicon (at 2.6° in 2theta) which was used as an internal scattering standard.^22^

Our interpretation of the cause of temperature drops upon recrystallisation of the amorphous ZIF-8, is supported by an experiment in which no recrystallization took place and consequently, a temperature drop was not observed after the steady-state temperature was reached (Fig. 4).

**Fig. 5:**
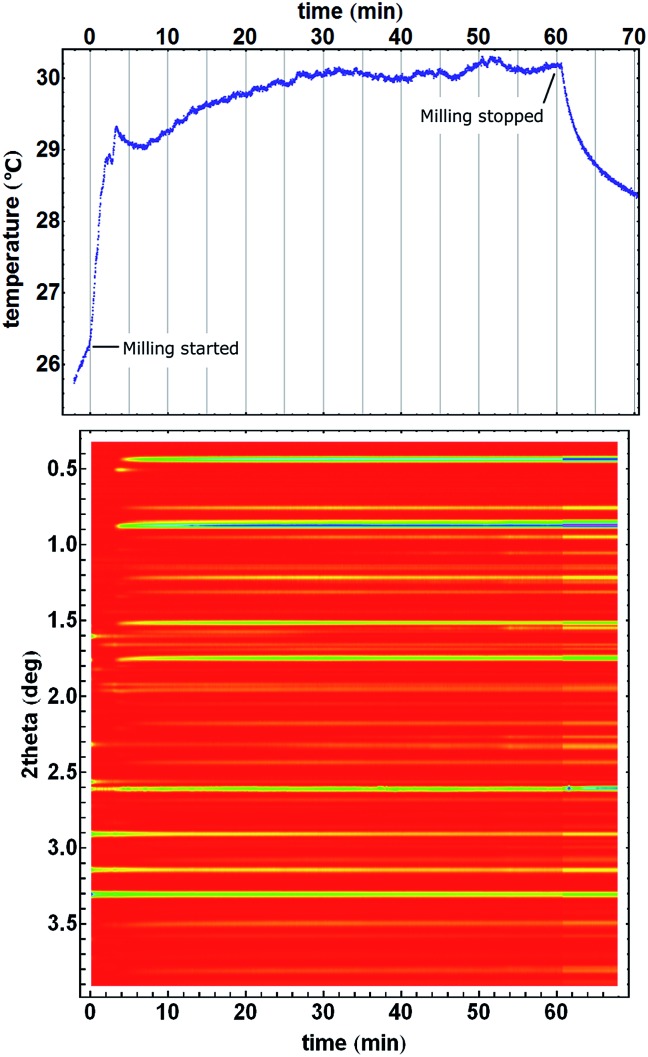
Mechanochemical formation of a pillared MOF material. Starting from ZnO, terephthalic acid, and dabco (1,4-diazabicyclo[2.2.2]octane),^50^ the formation of a fleeting short-lived intermediate (X-ray reflection at 0.5° in 2theta) around 4 minutes of milling can be observed in the temperature profile as an interruption in the heating profile.

The ZIF-8 → *kat* → *dia* polymorphic transformations was suitable to study reaction temperature profiles since the reaction mixture is always in the form of a free-flowing powder which ensures a reasonably uniform distribution of the reaction mixture inside the reaction vessel and a good thermal contact between it and the temperature sensor. However, we have also monitored the formation of other MOF materials. Mechanochemical formation of a pillared MOF^50^,^51^ exhibited pronounced jumps in the temperature profile (Fig. 5). After milling commenced, the temperature of the reaction mixture rose by almost 3 °C in only two minutes, followed by a short interruption in steady heating simultaneously with the formation of an intermediate phase that lived for one and a half minutes. The temperature stabilized at 29.8 °C after 30 minutes of milling, when the mechanochemical formation of pillared MOF was finished. This steady state, with minor deviations (±0.2 °C), remained during the next 30 minutes, until milling stopped and the milling assembly began to cool. We also noted that in some cases of sticky reaction mixtures, the temperature profiles may exhibit features that poorly correlate with composition changes but rather to the variations in a non-uniform distribution of the material inside the vessel (ESI Fig. 6†).

## Conclusions

We have presented here the first method for precise temperature monitoring of the sample during a mechanochemical reaction, and coupled it in tandem with *in situ* synchrotron powder X-ray diffraction, to reveal unexpected temperature profiles which correlate with changes in the reaction mixture. These unexpected temperature profiles in which the reaction mixture cools during slightly exothermic transformations, were explained by numerical simulations of heat flow during milling, which revealed the dominant influence of frictional heating to the observed temperature profiles, with the contributions of reaction enthalpies being significantly smaller and even negligible. Specifically, we showed by experiments and modelling that changes in reaction mixture composition due to the formation of new materials are found to result in changes to frictional properties of the milled mixture, which permit the thermal detection of transformations with even very small reaction enthalpies. The results presented here offer a new perspective to understand the development of thermal effects during milling and also provide a novel method to use temperature in monitoring mechanochemical reactions, even when enthalpy contribution may be too small to be of significance.

## Experimental

### Tandem *in situ* temperature and X-ray diffraction reaction monitoring

Milling experiments were performed using a modified Retsch MM301 ball mill operating at 30 Hz. The modification to the mill allowed for the X-ray beam (300 μm in diameter) to pass through the mill and through the bottom of the inside reaction vessel. A special reaction vessel was manufactured which had an embedded resistance temperature sensor (RTD) Pt-100 that was placed in thermal contact with a small piece of aluminum (aluminium plug) which was in direct contact with the reaction mixture (ESI Fig. 1 and 2†). In this manner, we have avoided collisions of the milling balls with the temperature sensor while still having a good thermal contact between the sensor and the reaction mixture. The reaction vessel was equipped with electronics which transformed the RTD readings into a digital signal which was sent to a computer for logging *via* an infrared transmitter. Electronics were powered by a set of batteries and was autonomous. Temperature readings were typically collected once every second. To ensure reproducibility and transferability of previous *in situ* monitoring experiments, the reaction vessel was manufactured to have the same internal volume of 14 mL and two stainless steel balls of 7 mm in diameter (mass of 1.4 g) were used as milling media.^25^

Tandem *in situ* experiments were performed at the old ID15B beamline of the European Synchrotron Radiation Facility (ESRF) in Grenoble, using high-energy monochromatic radiation (*E* = 88.0 keV, *λ* = 0.141 Å). Simultaneously with temperature monitoring, the course of studied reactions was monitored using X-ray diffraction of high-energy synchrotron radiation as described previously.^20^ The X-ray beam was passed through the lower part of the reaction vessel and the diffraction data were collected typically every 6 seconds on a flat-panel two-dimensional X-ray detector from Perkin-Elmer. The X-rays were selected using a bent double silicon monochromator. The X-ray wavelength and detector distance were calibrated using the NIST CeO_2_ standard sample which was packed in a capillary and was positioned at the bottom inside of the reaction vessel. Calibration and radial integration of raw diffraction images was performed using the program Fit2D (ESRF Internal Report, ESRF98HA01T, FIT2D V9.129 Reference Manual V3.1, 1998). Time-resolved diffractograms were plotted using the program Mathematica, using diffraction patterns with the background removed using the Sonneveld–Visser algorithm.^53^

### Simulations of temperature profiles

A 3D model of the vessel was made in the software package SolidWorks and simulations of temperature profiles were performed in SolidWorks Simulation module. Geometry of the model corresponded to the actual reaction vessel and is shown in ESI Fig. 7.† Materials used in simulation were PMMA for the reaction vessel and aluminium for the plug which is in contact with the temperature sensor. Since the plug was embedded in the vessel, the contact between these two components was set to *bonded*.

Two boundary conditions were defined for the inner surfaces of the vessel; zero heat flux density along the two planes of symmetry and the given amount of total heat flux on the surface that is in contact with the milling balls and the reaction mixture. Boundary conditions on vessel outer surfaces were set according to Newton's law of cooling with a heat transfer coefficient of 15.5 W m^–2^ K^–1^ and the bulk temperature equal to the simulation initial temperature.

Since the materials of the aluminium plug and the vessel differ, the density of the mesh was increased in the contact zone. The mesh (ESI Fig. 7†) was created in high quality (element size is 0.5 mm) with standard Voronoi–Delaunay meshing scheme and it consists of total 440 003 parabolic tetrahedral solid elements. The transient iterative solver was used with 10 s time step.

Since the temperature variations on the plug surface inside the vessel were within 2 mK, resulting output was an average temperature on the aluminium plug surface for every time step.

## Author contributions

Project was conceived by KU and supervised by KU, IH and TF. Temperature-measuring device was built by TM. Experiments were performed by KU, TM, IH, PAJ and TF. Heat flow was analysed by NF and BH. Numerical simulations were performed by NF. Figures were prepared by IH and NF. IH wrote the initial draft of the manuscript. All authors discussed the results and contributed to the final preparation of the manuscript.

## Conflicts of interest

There are no conflicts to declare.

## Supplementary Material

Supplementary information
