# Supplementary material for: Enthalpy vs. friction: heat flow modelling of unexpected temperature profiles in mechanochemistry of metal–organic frameworks
Source: Chem Sci. 2018 Jan 23;9(9):2525–32. doi: 10.1039/c7sc05312f (PMC5911823; doi:10.1039/c7sc05312f)
Supplement: Supplementary file 1 [file SC-009-C7SC05312F-s001.pdf]

Electronic Supplementary Material (ESI) for Chemical Science.
This journal is © The Royal Society of Chemistry 2018

Supplementary Information

for

# Enthalpy vs. friction: Heat flow modelling of unexpected temperature profiles in mechanochemistry of metal-organic frameworks

**S1. Temperature measurements**

Device:

A resistance temperature detector (RTD) Pt-100 was placed in thermal contact with the piece of aluminum which was in direct contact with the reaction mixture. In this manner we have avoided direct impact of the milling balls with the temperature sensor. The reaction vessel was also equipped with custom-designed electronics which transformed the RTD readings into a digital signal which was sent to a computer for logging *via* an infrared transmitter. The electronics was powered by a set of batteries. Our reaction vessel was thus autonomous and required no wires for operation. Temperature readings were typically collected once every second.

**Figure S1. Standard deviation of temperature readings is estimated from a long measurement where the temperature was constant.**

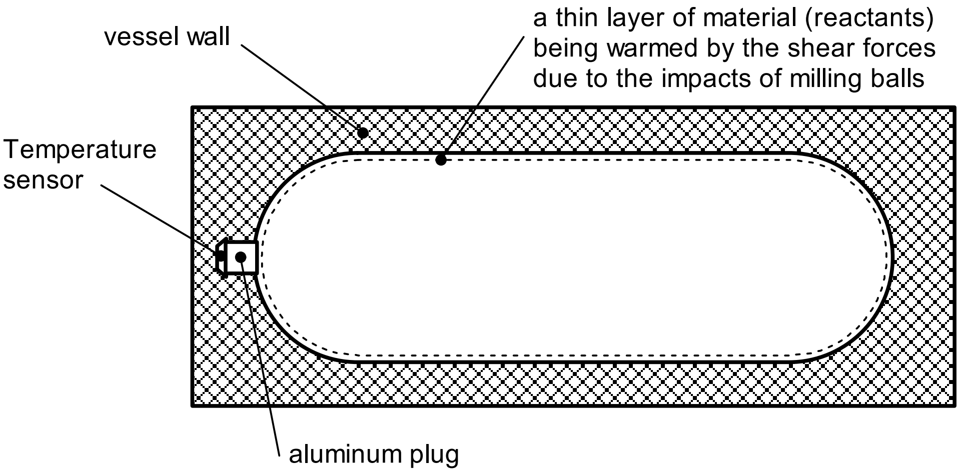


**Figure S1. The PMMA milling reaction vessel devised for in situ temperature monitoring of the bulk reaction mixture during milling.** The vessel is equipped with an aluminum plug and an RTD sensor (Pt-100), and logs the measured data by an infrared transmitter.

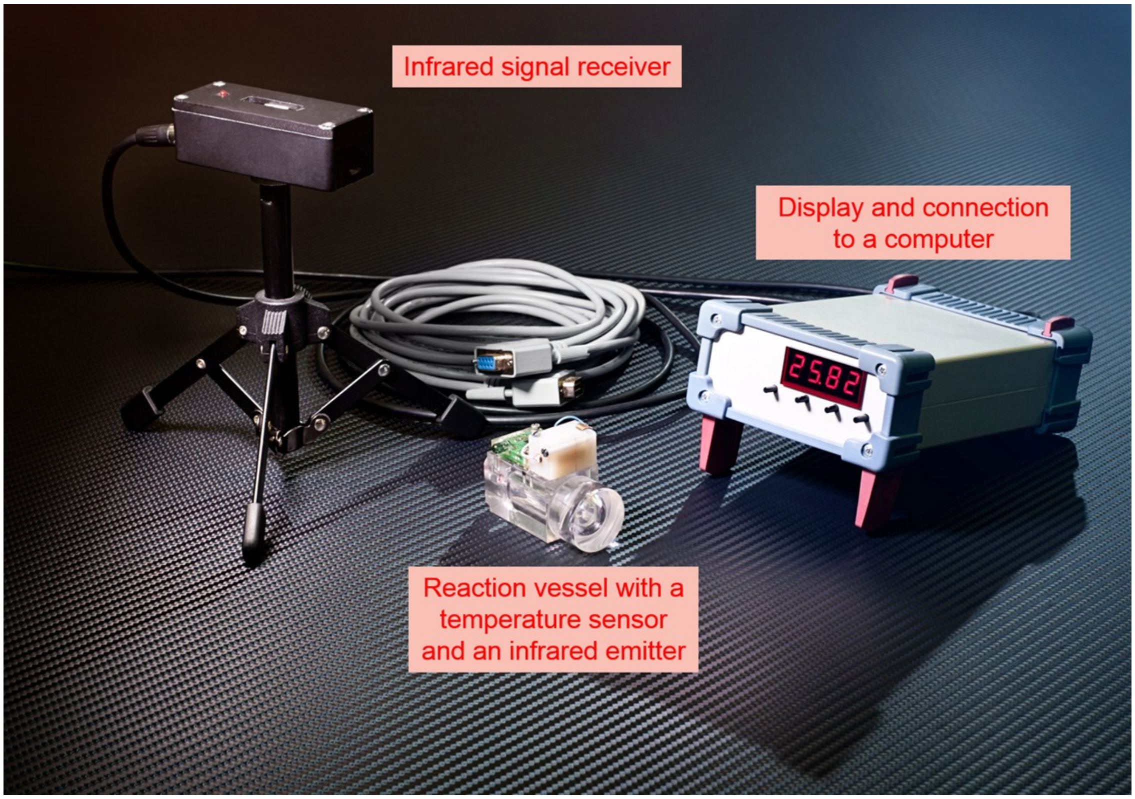


**Figure S2. Set-up for temperature monitoring**.

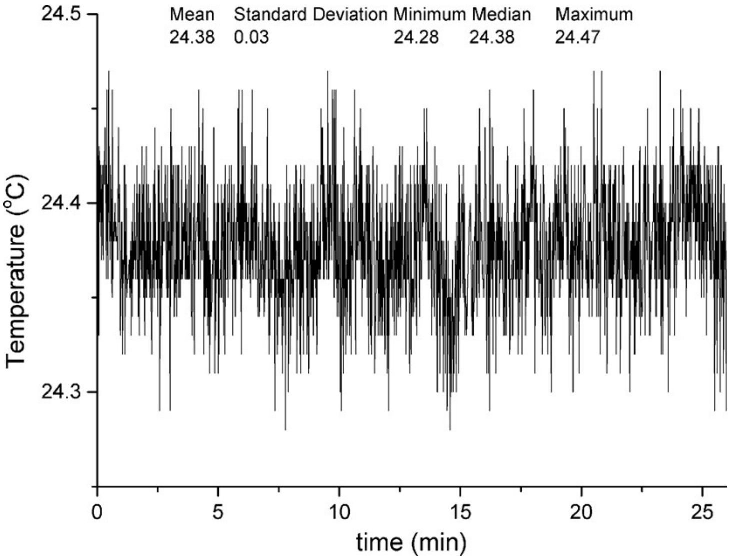


**Figure S3. Standard deviation of temperature readings is estimated from a long measurement where the temperature was constant.** Standard deviation of thus collected temperature readings is 0.03 °C.

Due to the very intensive and random movements of the balls within the vessel and layer redistribution over the surface as a consequence, it can be assumed that within a reasonably short time interval, the temperature of reacting material in the vessel is uniform and as such is simultaneously increased in time.

So, the part of the layer covering the aluminum plug inner surface can be assumed to represent the temperature of the whole reacting material. Since aluminum has extremely high thermal conductivity ($\lambda \cong 200$ W $m^{-1}$ $K^{-1}$) and very high *thermal diffusivity* $a = \lambda/(c\ \rho)$ ($\lambda \cong 200$ W $m^{-1}$ $K^{-1}$; $c \cong 900$ J $kg^{-1}$ $K^{-1}$; $\rho \cong 2700$ kg $m^{-3}$; $a \cong 8{,}5 \cdot 10^{-5}$ $m^2$ $s^{-1}$), and the plug is short (few millimeters) in the direction of heat conduction, it can be reasonably assumed that in any instant the outer end temperature of the plug is practically equal to its inner end temperature. The RTD sensor is thus able to precisely register *without delay* the *value* which can be assumed to represent the temperature of the reactants at any reasonably short time interval during the milling process.

Alternative temperature measuring methods like reading the temperature of the outside surface of the vessel (either by a contact probe or by an infrared camera) may register the outside temperatures which are both delayed by several seconds up to few minutes relative to the inside temperature and of different (smaller) magnitude, depending on the thickness of the vessel wall and the wall properties (thermal diffusivity).

## S2. **Analysis of heat exchange during milling**

The milling process starts with all the participants (reactants, vessel, and balls) at (approximately) the ambient temperature (the given *initial conditions*). After putting the vessel into a mill grips, the mill is started and the oscillatory (approximately axial) movements of the vessel begin.

The inertia of the balls and the quick moving of the vessel make the balls impact the semispherical ends of the cavity or slide along the length of the cavity. Wherever the impact takes place , the loss of kinetic energy due to non-ideal elastic deformation as well as the friction forces of the material being crushed between the balls and the vessel inner surface make the reacting material and the vessel inner surface warm-up. We will refer to the described phenomena as *friction* further in the text. As the temperature of the reactants layer rises, more heat is transferred to the vessel inner surface and through the wall to its outer surface and eventually to the ambient.  This is the non-steady state process but if the milling lasts long enough without the change of the ambient conditions, each part of the system tends asymptotically to reach its final, steady-state temperature.

During the mechanochemical process there are two energy-active phenomena – the *friction* and the *enthalpy of reaction* if and when the chemical reaction or the phase change takes place.

The milling is the *continuous* action during the whole mechanochemical process and is achieved by the violent shaking of the milling vessel and the random movement of the milling balls within it and relative to the vessel walls. It is *always* a *heat-release* process based on the impacts, friction and crushing of the material (later simply referred to as *friction*). The dissipative nature of the friction of any kind makes a part of the kinetic energy of the impacts of balls to the walls transform into an internal energy increase of the participants. The amount of energy dissipated depends on the balls-to-vessel-wall movement and on the milled material properties. If there were no material in the vessel, the agitation would “produce the heat energy” only due to the impact of balls into the vessel wall and due to the sliding/rolling friction between the wall and the balls and it is, assumedly, of relatively smaller intensity than when the material is present.

The enthalpy of reaction is released/absorbed during the chemical reaction, depending on the exothermic/endothermic nature of the reaction, and is superimposed as a positive/negative value to the (always positive) *frictional* heat. However, it is of *limited duration* and is present only during the reaction itself. Because of the small amount of reactants within the vessel, this amount of energy is usually also small compared to the energy released by the continuous *friction* process.

Both the energy dissipated by *friction* and the enthalpy of reaction are intrinsic to the interior of the milling vessel. As a consequence, the heat is transferred to the cooler vessel wall (heating it up) and finally to the ambient.

Since both *frictional* heat source and the reaction heat source/sink are of rather small intensity as compared to vessel wall heat capacity, the vessel wall temperature rises slowly and it may take the temperature even an hour to rise by only several Kelvins!

The milling process at any instant, or for the whole duration $t$ [s] from the beginning to the end, obeys the First Law of Thermodynamics (Energy conservation principle). For the milling vessel as a system, we can write:

$$Q_{in} + Q_{out} = U_2 - U_1 \quad \text{(S.1)}$$

where:

- the "heat input" $Q_{\text{in}}$ (from inside to the inner wall) is due to the *friction* and possible chemical reactions or phase changes. This term can be considered to be (at least approximately) independent on the inner temperature;

- the heat output $Q_{\text{out}}$ from the outer vessel wall to the ambient is due to the convection and radiation. According to the Newton's law of cooling, a convective heat flux density transferred to the ambient depends on the product of heat transfer coefficient and a temperature difference between ambient temperature $T_{\text{amb}}$ and the outer vessel temperature $T_{\text{out}}$. Radiation heat transfer, on the other hand, is known to be nonlinear function of ambient and outer vessel temperatures. But for small temperature differences (which is usually the case in mechanochemical experiments), radiation heat flow can be linearized and to a great accuracy can be considered as a function of temperature difference ($T_{\text{amb}} - T_{\text{out}}$). So, the magnitude of heat output $Q_{\text{out}}$, either (usually) negative/output or (less likely) positive/input, is determined by the difference ($T_{\text{amb}} - T_{\text{out}}$) between the ambient temperature $T_{\text{amb}}$ and the vessel outer temperature $T_{\text{out}}$, and by the parameters that influence the convection and radiation heat transfer (convective heat transfer coefficient, radiative properties etc.);

- the internal energy *increase in time* $U_2 - U_1$ is accumulated in the vessel wall due to the difference between the heat input from the inside and the heat output to the outside. The distribution of temperature within the wall is dictated by the Fourier's law of conduction imposed by the heat transfer through the vessel wall. In the steady state, the temperature field in the vessel body becomes constant in time and the accumulated energy in the vessel body is constant in time too.

The equation (S.1) can be written as:

$$Q_{friction} + Q_{reaction} + Q_{out} = U_2 - U_1. \quad \text{(S.2)}$$

The energy flow rate, $\dot{E}_{\text{f}}$ (W), that is transformed from the kinetic energy into the internal energy of the layer of reactant material by *friction* can be expressed as:

$$\dot{E}_f \simeq \dot{n}\, \dot{E}\, (1 - e^2). \quad \text{(S.3)}$$

where

- $\dot{n}$ is the average unit time number of impacts of the balls into the vessel wall. It depends on the milling parameters (amplitude and frequency of the oscillatory movement of the vessel), but does not depend on the temperature of the material within the vessel;

- $\grave{E}$ is the mean energy of the impact (depends on the balls velocity relative to the vessel inner surface lined with the material) and should not depend on the material layer temperature;
- *e* is the "coefficient of restitution" of the ball during impact with the vessel wall. It was experimentally determined to be approximately 0,3 for the impact of the steel ball onto a PMMA surface lined with a 1 mm thick reactant layer. Depending on the layer's internal friction, it is not likely to be highly affected by the relatively small temperature change of the reactants temperature (about five degrees Celsius) during the milling and therefore can be considered constant.

Due to a random movement of balls within the vessel, the dissipated energy flow rate $\dot{E}_f$ is clearly of stochastic nature This means that dissipated energy cannot be calculated from a single ball impact since a number of impacts are unknown. However, because of very high milling frequency, a number of impacts within a *reasonably short time interval* is high enough that can be represented with an average number of impacts which is approximately equal for every such interval. This means that dissipated energy flow rate will be approximately equal for every reasonably short time interval and according to S.3 approximately independent of temperature.  For further analysis, without loss of generality, its mean value per unit time will be substituted and treated as continuous variable.

The energy flow rate $\dot{E}_f$, dissipated by friction in the reactant layer, is transferred as the heat flow $\Phi_{friction}$ to the cooler vessel inner wall increasing its temperature in time. However, the heat flow rate $\Phi_{friction}$, independent of the increasing layer's temperature, remains unchanged as long as the layer's material and the milling parameters are the same. It is important to emphasize that this doesn't mean $\Phi_{friction}$ is constant during the whole milling process, because a chemical reaction or crystalisation of the reactants can change the *frictional* properties inside the vessel. If *frictional* properties are the same during the whole mechanochemical process, a simple relation is valid: $Q_{friction} = \Phi_{friction} \times t$. Otherwise, $Q_{friction}$ is the sum of *frictional* energies dissipated within each time period of constant *frictional* properties within the vessel.

The rate $\Phi_{reaction}$ at which the heat of reaction $Q_{reaction}$ is released or absorbed depends on the kinetics of the reaction and is usually unknown, but the total amount of energy is known to be $m_{reactants} \times \Delta h_{reaction}$.

The equation (S.2) can be written also for an infinitesimal time step (assumedly inside the duration of the reaction):

$$\Phi_{friction}(t)\,dt + \Phi_{reaction}(t)\,dt + \Phi_{out}(T_{out}, t)\,dt = \frac{dU(T_{wall}, t)}{dt}\,dt, \qquad (S.4)$$

showing that the vessel's wall temperature change in time can be influenced by the reaction enthalpy during the reaction.

However, the integration over a long period of time, *t*, (embracing the whole possible chemical reaction or phase change duration)

$$\underbrace{\int_{t=0}^{t}\Phi_{\text{friction}}(t)\,\mathrm{d}t}_{\text{increases with time}} + \underbrace{m_{\text{reactants}}\Delta h_{\text{reaction}}}_{\text{remains constant}} + \underbrace{\int_{t=0}^{t}\Phi_{\text{out}}(T_{\text{out}},t)\,\mathrm{d}t}_{\text{increases with time}} = \underbrace{\int_{t=0}^{t}\frac{\mathrm{d}U(T_{\text{wall}},t)}{\mathrm{d}t}\,\mathrm{d}t}_{\text{tends to a constant value for the whole process: } U_2-U_1} \tag{S.5}$$

shows that the second term on the left side of (S.4) is of constant value and becomes ever more negligible compared to the first term as the process approaches its steady-state (upper boundary of integration tends to $\infty$). Also the term ($\mathrm{d}U/\mathrm{d}t$), the *time-change* of the internal energy of the vessel wall, will vanish when the steady-state is achieved making the righthand side of eq. S.5 time independent and equal to $U_2(T_{wall}, t = \text{infinity}) – U_1(T_{wall}, t = 0)$.

The integration in time from 0 to $\infty$ makes the two terms of limited amount negligible and yields the steady-state condition:

$$\Phi_{\text{friction}}(t \to \infty) + \Phi_{\text{out}}(T^*_{\text{out}}, t \to \infty) = 0 \tag{S.6}$$

where $T^*_{\text{out}}$ is the steady state temperature field of the vessel outer surface, closely related to the steady state inner surface temperature field $T^*_{\text{in}}$. Since the friction heat flow is *always positive*, the heat flow exchanged with the ambient in the steady state must *always* be *negative/output*, meaning that in the steady state the vessel's outer surface temperature (and even more so its inner temperature) must always be above the ambient temperature.

These relationships clearly show that the steady-state temperature of the apparatus does not depend on the reaction energy (enthalpy), but solely on the friction heat source intensity and the ability of the milling vessel to transfer this heat to the ambient. The reaction enthalpy can *temporarily* change the intensity of the heat source or even revert it to the heat sink, but the effect stops with the end of reaction/phase change and the dominant influence of the friction is reestablished. If, after the reaction is finished, a new asymptotic value of the steady-state temperature appears, this must be a result of different *friction* conditions within the vessel due to a change of parameters that, according to (S.3), influence energy flow rate $\dot{E}_f$. The newly formed product(s) must have different *frictional* properties than the reactants. Of course, not to be forgotten, the change in the steady-state temperatures can be the result of altered heat transfer conditions on the ambient side too, but for the continuous and controlled milling process this is not likely to be the case.

---

To check the validity of the above statements, several typical cases were analyzed by comparing numerical simulation results to the measured data:

a) the “milling” of the empty vessel (without material);

b) the milling of the inert material (ZnO);

These two cases are chosen because during both experiments a *frictional* properties and so a *frictional* heat flow rate, $\Phi_{\text{friction}}$, remain unchanged.

Ad a) the “milling” of the empty vessel (without material);

The comparison results in the case of empty vessel “milling” are shown on Figure 5. A very good match of simulation results with measured temperature response is obvious. In the simulation, a constant total heat generation rate that corresponds to a *frictional* heat flow rate inside the milling vessel was set to 272 mW. Almost perfect overlapping between these two temperature profiles, as stated earlier indicates that, when observing a *reasonably short time interval*, frictional heat flow rate, $\Phi_{\text{friction}}$, which cause a temperature rise, can be considered constant and temperature independent. Temperature profile shows steady-state temperature rise of about 4 °C which is determined by the heat flow rate, $\Phi_{\text{friction}}$ inside the vessel and the heat output according to the Newton’s law of cooling.

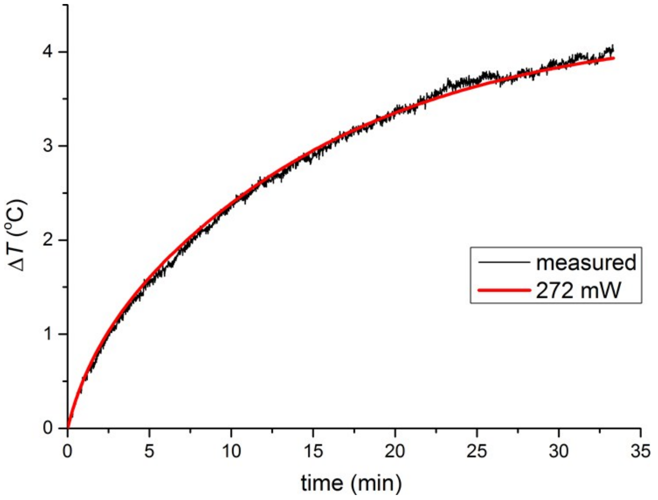


**Figure S4. Simulation of heat flow for an empty vessel.** Required heat flow rate generated by ball impacts to reproduce the observed heating profile is 272 mW.

Ad b) the milling of the inert material (ZnO);

The second example results are shown in the Figure 6.  Increasing a total heat flow rate in the simulation from 272 mW (in the case of empty vessel) to 496 mW yields a very good match of simulation and measurement results. This means that estimated *frictional* heat flow rate, $\Phi_{\text{friction}}$, inside the vessel is increased due to a different frictional properties when inert ZnO is present. The fact is confirmed by measuring coefficient of restitution which was lowered form 0.86 in the case of PMMA-ball impact to 0.3 in the case of PMMA-ZnO-ball impact. It means that dissipation rate of kinetic energy in the latter case is higher, so that more heat is transferred to the material and vessel surfaces. This is the reason why steady-state temperature rise is increased to ca. 5.5 °C.

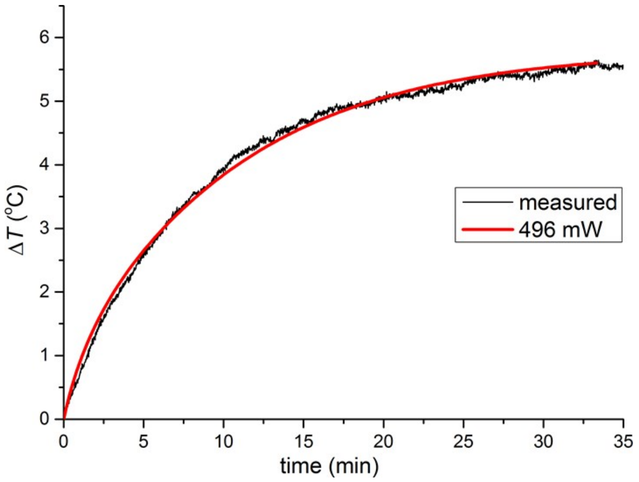


**Figure S5. Simulation of heat flow for milling of ZnO, a material that does not noticeably change upon applied milling conditions**. The required heat flow rate generated by ball impacts to reproduce the observed heating profile is 496 mW.

Presented analysis proves that the steady-state temperature, besides ambient conditions, is influenced by the frictional properties in the vessel. These frictional properties can be changed during mechanochemical process if the new substances or phases are produced, making the temperature in the vessel approach the new steady state temperature. The short term disturbances like reaction energies or phase changes can only cause temporary deviations from temperature profile that is determined by ambient conditions and frictional properties in the vessel. By knowing the parameters that influence a steady-state temperature, from observed temporary deviations one can conclude about chemical processes occurring in the vessel.

## S3. Non-uniform distribution of the material the reaction vessel during milling

The milled mixture may suffer from non-uniform distribution in the reaction vessel. This is particularly likely in the case of milling of sticky reaction mixtures (Figure S4). Sticky reaction mixtures may result from transformation of porous materials into non-porous materials where the pore content (often liquids) needs to exit the pores leading to wet milling. In such instances, the temperature profile may reflect more the distribution of the material inside the vessel rather than any chemistry occurring during milling.

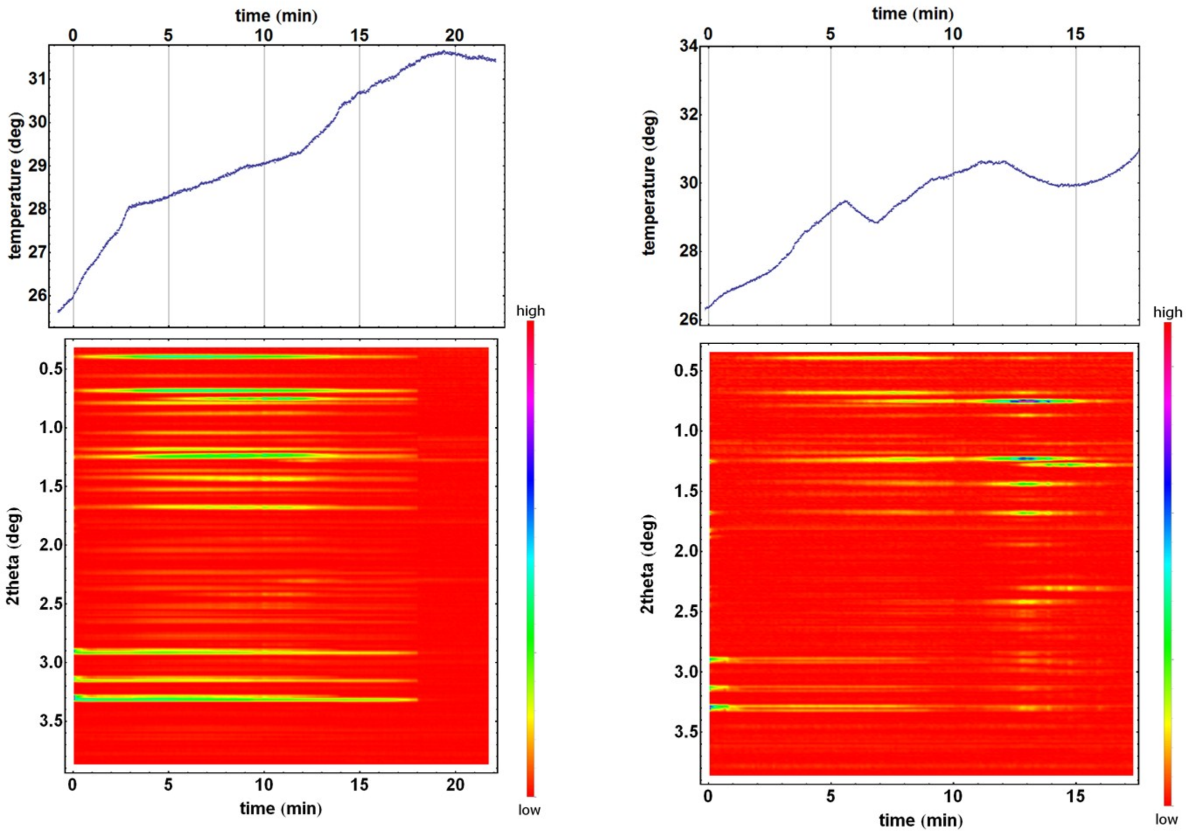


**Figure S6. Staring from the same initial conditions, the same milling experiments resulting in different kinetics and temperature profiles.** Mechanochemical LAG preparation of the tetragonal pillared MOF from ZnO, terephthalic acid and dabco (1,4-diazabicyclo[2.2.2]octane). In both experiments the reaction mixture was sticky resulting in a non-uniform distribution of the material inside the reaction vessel. The temperature profile is thus difficult or impossible to interpret in terms of the chemical transformations that are occurring.

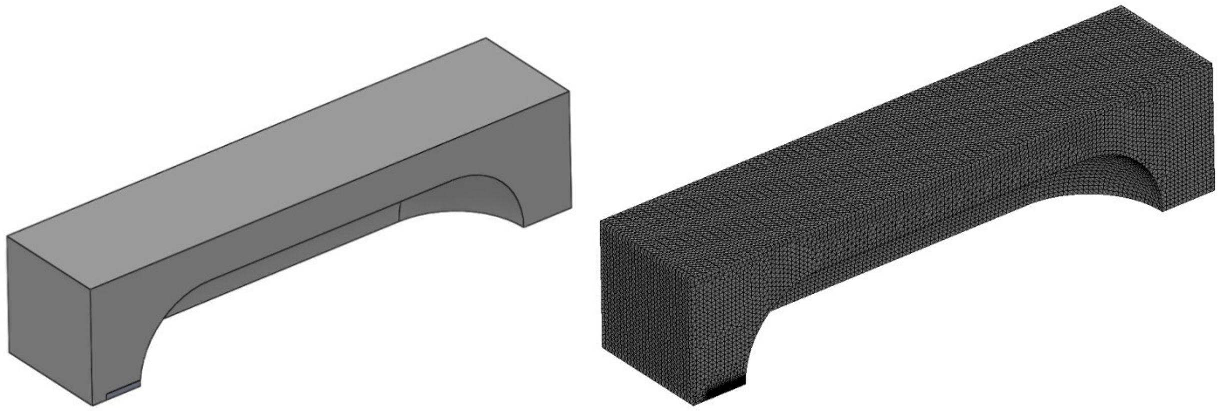

**Figure S7. Reaction vessel model**. (left) 3D model and (right) mesh.
